# Effect of occlusal adjustment on post-orthodontic relapse in extraction cases: a randomized clinical trial

**DOI:** 10.21142/2523-2754-1402-2026-290

**Published:** 2026-04-04

**Authors:** Arezoo Jahanbin, Maryam Omidkhoda, Farnaz Zia, Azam Sadat Madani, Ali Koohrokhi, Mona Kazemi

**Affiliations:** 1 Department of Orthodontics, School of Dentistry, Mashhad University of Medical Sciences. Mashhad, Iran. jahanbinA@mums.ac.ir , maryamomidkhoda@gmail.com, monakazemi.dnt@gmail.com, madania@mums.ac.ir, akoohrokhi@gmail.com Mashhad University of Medical Sciences Department of Orthodontics School of Dentistry Mashhad University of Medical Sciences Mashhad Iran jahanbinA@mums.ac.ir maryamomidkhoda@gmail.com monakazemi.dnt@gmail.com madania@mums.ac.ir akoohrokhi@gmail.com; 2 Department of Orthodontics, School of Dentistry, Guilan University of Medical Sciences. Rasht, Iran. farnaz_ziya91@yahoo.com Gilan University of Medical Sciences Department of Orthodontics School of Dentistry Guilan University of Medical Sciences Rasht Iran farnaz_ziya91@yahoo.com

**Keywords:** orthodontics, orthodontic relapse, occlusal adjustment, ortodoncia, recidiva ortodóncica, ajuste oclusal

## Abstract

**Objectives:**

Maintaining post-treatment occlusal stability remains a major challenge in orthodontics. Premature occlusal contacts may contribute to relapse. This study aimed to evaluate the effect of selective occlusal adjustment on relapse six months after debonding.

**Materials and Methods:**

Thirty Class I patients (17-28 years) with severe crowding, treated with fixed appliances and extraction of first premolars, were randomly assigned to an intervention group (occlusal adjustment one month after debonding) and a control group (no adjustment). Relapse was assessed on dental casts immediately post-debonding and six months later, measuring inter-canine width, intermolar width, arch length, and Modified Little’s Irregularity Index. Data were analyzed using independent and paired t-tests, Mann-Whitney, Wilcoxon, and chi-square tests (α = 0.05).

**Results:**

This study demonstrated that after occlusal adjustment, the maxilla showed significant increases in intermolar width, intercanine width, and arch Circumference compared with the controls after six months post-debonding (p = 0.009, 0.011, 0.041). In the mandible, the changes in intermolar width and arch Circumference were significantly greater than those in the control group (p = 0.037 and p = 0.010, respectively), and the irregularity index in the intervention group decreased in the maxilla and increased in the mandible compared to the control group; however, these changes were not significant in either arch.

**Conclusion:**

Selective occlusal adjustment may enhance transverse arch stability after orthodontic treatment; however, its isolated effect on anterior alignment relapse remains inconclusive.

## RESUMEN

**Objetivos:**

Mantener la estabilidad oclusal postratamiento sigue siendo un reto importante en ortodoncia. Los contactos oclusales prematuros pueden contribuir a la recidiva. Este estudio tuvo como objetivo evaluar el efecto del ajuste oclusal selectivo sobre la recidiva seis meses después del descementado de brackets.

**Materiales y métodos:**

: Treinta pacientes de Clase I (17-28 años) con apiñamiento severo, tratados con aparatología fija y extracción de primeros premolares, fueron asignados aleatoriamente a un grupo de intervención (ajuste oclusal un mes después del descementado) y a un grupo control (sin ajuste). La recidiva se evaluó en modelos dentales inmediatamente después del descementado y seis meses después, midiendo la anchura intercanina, la anchura intermolar, la longitud de arcada y el Índice de Irregularidad de Little Modificado. Los datos se analizaron mediante pruebas t independientes y pareadas, Mann-Whitney, Wilcoxon y Chi-cuadrado (α = 0,05).

**Resultados:**

Este estudio demostró que, tras el ajuste oclusal, el maxilar mostró aumentos significativos en la anchura intermolar, la anchura intercanina y la circunferencia de la arcada, en comparación con el grupo control, seis meses después del descementado (p = 0,009, p=0,011, p=0,041). En la mandíbula, los cambios en la anchura intermolar y la circunferencia de la arcada fueron significativamente mayores que en el grupo control (p = 0,037 y p = 0,010, respectivamente), y el índice de irregularidad en el grupo de intervención disminuyó en el maxilar y aumentó en la mandíbula, en comparación con el grupo control; sin embargo, estos cambios no fueron significativos en ninguna de las arcadas.

**Conclusión:**

El ajuste oclusal selectivo puede mejorar la estabilidad transversal de la arcada tras el tratamiento de ortodoncia; sin embargo, su efecto aislado sobre la recidiva de la alineación anterior no es concluyente.

## INTRODUCTION

Orthodontic relapse, commonly defined as the tendency of teeth to return toward their pre-treatment positions following active treatment, remains a significant challenge in achieving long-term treatment success ^1^^,^^2^. Beyond simple positional changes, relapse can encompass any post-treatment deviations from the optimal occlusal relationships established at the end of active therapy. Ensuring occlusal stability is thus a fundamental goal of orthodontic treatment, as instability may compromise both functional and esthetic outcomes ^3^.

One of the proposed contributors to post-treatment tooth movement and relapse is the presence of premature occlusal contacts ^4^. These contacts generate eccentric lateral forces on the dentition, which may induce undesirable tooth movements such as tipping, rotation, or space opening ^5^^,^^6^. Consequently, occlusal adjustment, defined as the selective modification of occlusal contact points, has been suggested as a means to redistribute occlusal forces more evenly, thereby reducing the risk of relapse ^7^.

Since Andrews introduced the Six Keys to Normal Occlusion in 1972, these principles have provided a standardized framework for assessing post-treatment occlusal quality ^8^. Nevertheless, even after comprehensive orthodontic correction, residual occlusal interferences may persist and potentially undermine long-term stability. Such interferences can be addressed through occlusal equilibration procedures, including selective grinding, which aims to refine contacts and improve functional occlusion ^6^. Despite this, orthodontists are advised to prioritize achieving optimal occlusal finishing during active treatment to minimize the need for extensive post-treatment adjustment. Minor adjustments may be warranted when natural settling is insufficient, particularly if they enhance occlusal function and stability by modifying cusp morphology, fossa depth, or axial inclinations ^9^.

Various methods have been employed to evaluate occlusal contacts and interferences, ranging from qualitative techniques such as articulating paper and occlusion sprays to quantitative digital analyses. The accuracy and reliability of these assessment tools are critical in identifying true occlusal discrepancies that may contribute to relapse ^10^.

In the study by Edman Tynelius et al. ^11^, relapse-related changes across three retention protocols were evaluated at four time points. Their findings demonstrated that, in all three groups, the Little Irregularity Index decreased immediately after treatment but showed progressive increases at the 2- and 5-year follow-ups, particularly in the mandibular arch. In the mandible, intercanine width increased immediately post-treatment, exhibited a slight additional increase at two years, and then decreased at five years, whereas in the maxilla, an initial increase was followed by a gradual reduction over subsequent years. Mandibular intermolar width showed an immediate reduction, increased at two years, and then declined again at five years; in contrast, maxillary intermolar width remained essentially unchanged from pretreatment values but decreased at later follow-ups. Arch length in both arches decreased immediately after treatment, increased modestly at the two-year evaluation, and then declined again by the five-year mark.

However, the role of post-treatment occlusal adjustment in preventing relapse remains controversial. While several studies have examined occlusal changes during retention and the impact of fixed or removable retainers on stability, there is limited high-quality evidence directly linking occlusal adjustment to reduced relapse rates. For instance, occlusal adjustment has shown promise in enhancing stability in certain malocclusions, such as anterior open-bite cases ^12^. Conversely, long-term cohort studies have reported minimal or statistically insignificant benefits of occlusal equilibration on alignment stability, highlighting inconsistencies in the literature ^13^.

Given these conflicting findings, further clinical research is needed to clarify the impact of occlusal adjustment on post-treatment stability. This randomized clinical trial aimed to determine whether selective occlusal adjustment performed after debonding can reduce short-term orthodontic relapse six months after debonding. Should a significant difference be observed, occlusal adjustment may be considered a preventive adjunct in post-treatment protocols. Considering its low invasiveness, this approach could also offer a cost-effective strategy for minimizing long-term re-treatment expenses.

## MATERIALS AND METHODS

### Study Design and Sample Size Calculation

The study proposal was submitted and received approval from Mashhad University of Medical Sciences (IR.MUMS.DENT.REC.1401.143) and registered in the Iranian Registry of Clinical Trials (IRCT20230310057665N1). This randomized controlled trial included a pilot study with five patients per group. The most notable difference in pre- and post-intervention measurements was observed in Intermolar Distance (IMD), with means of 1.95 ± 1.80 mm in the intervention group and -1.0 ± 2.5 mm in the control group. An independent samples t-test showed a significant difference (p = 0.039). Based on these results, with 95% confidence and 80% power, the required sample size was 10 per group. To improve reliability, the sample size was increased to 15 patients per group, resulting in a total of 30 patients.

### Randomization

Block randomization was performed using PASS software to generate the allocation sequence, which was concealed using sealed opaque envelopes. Outcome assessors were blinded to group assignments, making this a single-blind study.

### Participants

Thirty orthodontic patients aged 17 to 28 years (17 females and 13 males) were recruited from the orthodontics department of the dental school clinic. All had Class I malocclusion with more than 8 mm of crowding based on Tooth Size-Arch Length Discrepancy. The Consolidated Standards of Reporting Trials (CONSORT) guidelines and the Helsinki Declaration principles were followed in this study. All participants provided informed consent.

### Inclusion Criteria

• Age 17-28 years
• Class I dental malocclusion with >8 mm crowding
• Undergoing fixed orthodontic treatment with the extraction of the four first premolars
• Treated using the MBT 0.022 fixed appliance system

### Exclusion Criteria

• Systemic diseases
• Poor oral hygiene (Silness and Löe Plaque Index ≥ 2)
• Gingival inflammation, periodontal disease, or deep caries
• Use of medications affecting bone metabolism
• Parafunctional habits or a history of temporomandibular joint disorders

### Study Groups

Participants were randomly assigned to two equal groups (n =15 each): 

*Control Group*, Alginate impressions (Study Model (SM0)) were taken immediately after debonding (T0), and follow-up impressions (SM1) six months later (T1). No occlusal adjustments were made.

*Intervention Group*, Initial impressions were taken immediately post-debonding (SM0). Selective occlusal adjustment was performed one month later, followed by follow-up impressions six months after debonding (SM1).

### Treatment Protocol

Following completion of orthodontic treatment with the MBT 0.022 system and extraction of four premolars, appliances and adhesives were removed, and teeth were polished. Alginate impressions (Chromogel, Marlik, Iran) were obtained, and casts were poured using Hinridur® S (Type III hard dental stone, manufactured by Ernst Hinrichs Dental GmbH, Germany).

### Retainer Protocol and Follow-up

Clear Essix retainers (1.5 mm thick) were fabricated and delivered within one week of post-debonding. Retainers extended 1-2 mm on the buccal and 3-4 mm on the palatal gingival surfaces. Patients were instructed to wear retainers full-time for four months (except during eating and brushing), then nightly thereafter ^14^.

### Occlusal Adjustment Procedure

**Figure 1:**
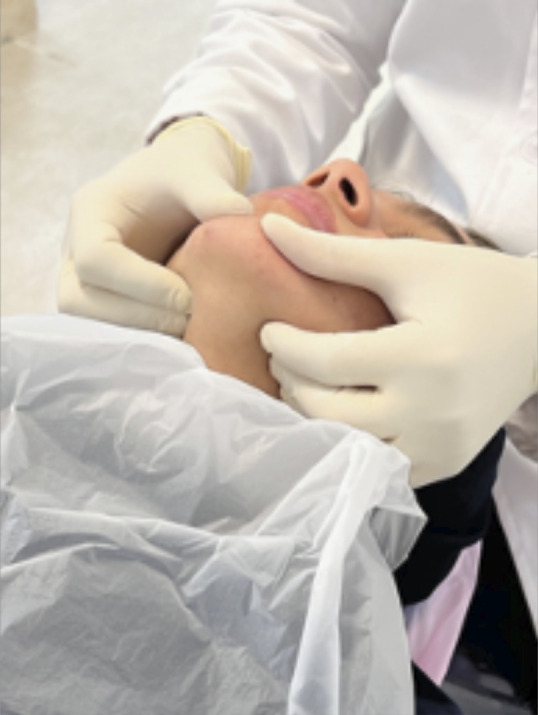
Mandibular positioning into centric relation (CR) using the Dawson Technique

At one month post-debonding, the intervention group underwent selective occlusal adjustment performed by a trained resident under supervision:

1. Patients were positioned supine with cotton rolls to relax muscles; mandibular positioning into centric relation (CR) was achieved using the Dawson technique (Figure 1) ^15^. 

2. Premature contacts in CR were marked using 8-µm green articulation tape (Arti-Fol, Germany).

3. Marked areas were selectively ground with an egg-shaped 833F bur (Jota, Switzerland).

4. Interferences during lateral and protrusive movements were identified and adjusted to establish canine guidance ^16^.

5. Adjusted surfaces were polished, and occlusion was verified for balance in centric and excursive positions.

### Measurement of Relapse

**Figure 2:**
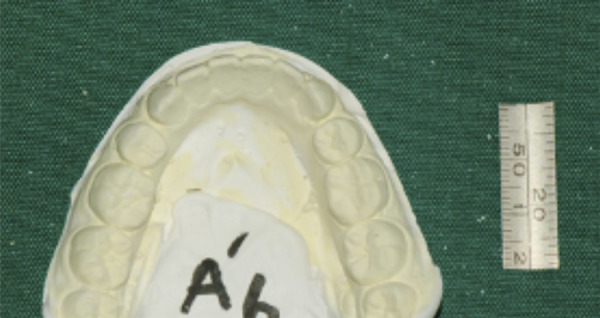
Casts photography with a Nikon camera (model 8010741) at a fixed vertical distance, calibrated using a measuring gauge beside the castse

**Figure 3:**
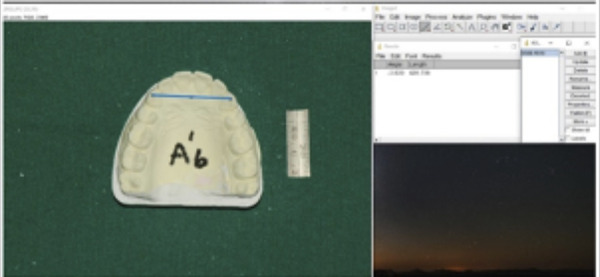
Intercaine Width- the distance between caine cusp tips

**Figure 4:**
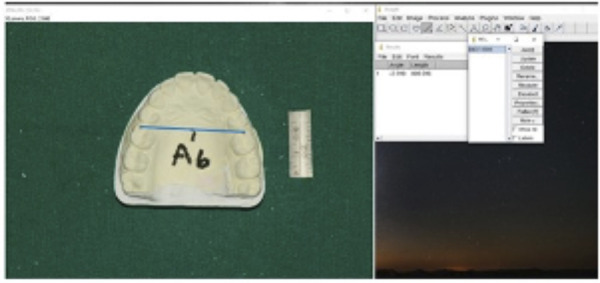
Intercaine Width- the distance between mesiobuccal cusp tips of first molars

**Figure 5:**
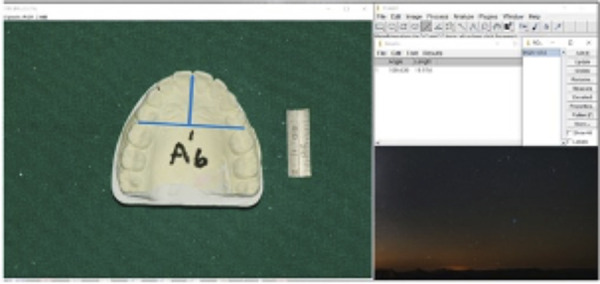
Arch Lenth- perpendicular distance from the central incisor contact point to the plane by the mesial marginal ridges of first molars

**Figure 6:**
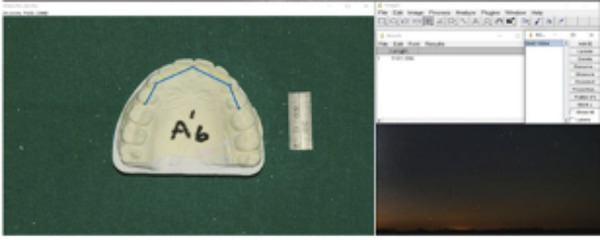
Arch Circumference- measurement tracing from first molar mesial contact through premolars and anterior teeth cusps

Casts were photographed using a digital single-lens reflex camera (Nikon D750, Nikon Corporation, Tokyo, Japan) at a fixed vertical distance, calibrated with a measuring gauge beside the casts (Figure 2). Digital images were analyzed using ImageJ software (NIH, USA) after magnification correction. The following parameters were measured: 

1. Intercanine Width: Distance between canine cusp tips (Figure 3)

2. Intermolar Width: Distance between mesiobuccal cusp tips of first molars (Figure 4)

3. Arch Length: Perpendicular distance from the central incisor contact point to the plane defined by the mesial marginal ridges of first molars (Figure 5)

4. Arch Circumference: Measurement tracing from the first molar mesial contact through the premolars and anterior teeth cusps (Figure 6)

5. Modified Little’s Irregularity Index: Sum of contact point displacements between adjacent anterior teeth from canine to canine 

### Statistical Analysis

Data distribution was assessed for normality. Parametric tests (independent and paired t-tests) were used for normally distributed data; non-parametric tests (Mann-Whitney U, Wilcoxon) were applied otherwise. Categorical data were analyzed using chi-square tests. Statistical significance was set at p < 0.05.

## RESULTS

A total of 30 orthodontic patients, including 17 females (56.7%) and 13 males (43.3%) aged 17 to 28 years (mean age 20.20 ± 3.20 years), were evaluated for intra-arch indices and relapse in two groups: intervention and control. The mean age was 20.57 ± 3.45 years in the intervention group and 19.83 ± 3.00 years in the control group, with no significant difference (p = 0.624, independent t-test). The gender distribution was also similar between the groups (p = 0.713, chi-square test), confirming baseline homogeneity (Table 1).

**Table 1:** Demographic variables in the control and intervention groups

Characteristics		Control Group	Intervention Group	P-Value
		Mean ± SD or n (%)	Mean ± SD or n (%)	
Age(year)		19.83 ± 3.00	20.57 ± 3.45	0.624*
Gender	Female	9(60.0)	8(53.3)	0.713**
	Male	6(40.0)	7(46.7)	

*Mann-Whitney test,

**Chi-square test

Measurements were taken at two time points: immediately after debonding (T0) and six months post-treatment (T1), for both the maxilla and mandible in each group. Normality tests indicated that most variables were normally distributed, informing the choice of parametric or non-parametric analyses. The results of the study are presented in Tables 2 and 3.

**Table 2:** Comparison of relapse variables in T0 and T1

Jaw	Variable	Intervention Group			Control Group		
		T0	T1	P-value	T0	T1	P-value
		Mean ± SD	Mean ± SD		Mean ± SD	Mean ± SD	
Maxilla	IMD	49.33 ± 1.56	51.30 ± 2.96	0.012*	48.93 ± 3.32	47.90 ± 3.26	0.157
	ICD	36.68 ± 2.04	37.95 ± 1.75	0.004*	36.71 ± 2.21	36.53 ± 1.62	0.639
	AL	22.03 ± 1.22	23.11 ± 0.88	0.008*	21.57 ± 2.49	22.00 ± 1.56	0.453
	AC	70.59 ± 3.46	73.41 ± 2.31	0.011*	69.85 ± 5.80	69.46 ± 3.25	0.740
	LII	4.23 ± 1.08	4.01 ± 0.88	0.126	4.20 ± 1.41	3.85 ± 1.24	0.410
Mandible	IMD	42.22 ± 2.08	43.80 ± 2.28	0.008*	42.67 ± 2.40	42.45 ± 3.60	0.820
	ICD	27.52 ± 1.54	27.81 ± 1.00	0.233	28.18 ± 1.13	28.44 ± 1.07	0.391
	AL	16.91 ± 1.61	17.36 ± 1.56	0.053	17.04 ± 1.96	17.02 ± 1.92	0.946
	AC	55.88 ± 3.61	58.20 ± 2.38	0.005*	57.13 ± 4.39	56.97 ± 4.38	0.838
	LII	3.90 ± 0.76	4.60 ± 0.88	0.007*	3.78 ± 0.89	4.32 ± 1.00	0.066

* Indicates statistically significant difference (p < 0.05)

**Table 3:** Comparison of relapse changes between groups

Variable	Maxilla			Mandible		
	Control Mean ± SD	Intervention Mean ± SD	P-value	Control Mean ± SD	Intervention Mean ± SD	P-value
IMD	-1.02 ± 2.64	1.97 ± 1.94	0.009*	-0.22 ± 3.69	1.58 ± 1.99	0.037*
ICD	-0.18 ± 1.45	1.27 ± 1.44	0.011*	0.26 ± 1.15	0.29 ± 0.82	0.951
Al	0.43 ± 2.16	1.08 ± 1.37	0.331	-0.02 ± 1.13	0.44 ± 0.82	0.208
AC	-0.39 ± 4.55	2.82 ± 2.74	0.041*	-0.15 ± 2.87	2.32 ± 2.68	0.010*
LII	-0.35 ± 1.60	-0.23 ± 0.54	0.713	0.54 ± 1.05	0.70 ± 0.86	0.650

* Indicates statistically significant difference (p < 0.05)

### 1. Intervention Group

- Maxilla: Significant increases were observed in the Intermolar Distance (IMD) (p = 0.012, paired t-test), Intercanine Distance (ICD) (p = 0.004), Arch Length (AL) (p = 0.008), and Arch Circumference (AC) (p = 0.011) after 6 months. The Little’s Irregularity Index (LII) showed a decrease; however, this change was not statistically significant (p = 0.126).

- Mandible: The IMD, AC, and LII significantly increased (p = 0.008, p = 0.005, and p = 0.007, respectively). The ICD and AL also increased, but without statistical significance (p = 0.233 and p = 0.053).

### 2. Control Group

- Maxilla: Mean values of IMD, ICD, AC, and LII decreased, though these differences were not significant (p = 0.157, 0.639, 0.740, and 0.410, respectively). Arch Length (AL) showed a non-significant increase (p = 0.453).

- Mandible: The IMD, AL, and AC decreased slightly but not significantly (p = 0.820, 0.946, and 0.838). Conversely, ICD and LII increased, although without statistical significance (p = 0.391 and p = 0.066).

### 3. Between-Group Comparisons

-Maxilla: Changes in IMD, ICD, and AC were significantly greater in the intervention group compared to the control group (p = 0.009, 0.011, and 0.041, respectively). Changes in AL and LII were higher in the intervention group but did not reach significance (p = 0.331 and 0.713).

- Mandible: The intervention group exhibited significantly greater changes in IMD and AC than the control group (p = 0.037 and 0.010). Changes in ICD, AL, and LII were higher in the intervention group but not statistically significant (p = 0.951, 0.208, and 0.650).

## DISCUSSION

Orthodontic relapse remains a major concern following active treatment, particularly in extraction cases, where the risk of post-treatment instability tends to be higher ^17^. Despite the widespread use of various retention protocols, the underlying etiologies of relapse are multifactorial and not fully understood ^18^. Among potential contributing factors, occlusal interferences have received limited attention in the orthodontic literature, despite being well-established in prosthodontics and restorative disciplines ^19^.

This randomized clinical trial evaluated the short-term (six months) effects of selective occlusal adjustment on changes in arch dimensions and anterior alignment among patients treated with fixed appliances and the extraction of four premolars, after treatment.

Six months after debonding, the intervention group showed significantly greater increases in maxillary intermolar width, intercanine width, and arch circumference compared with the control group. In the mandible, a similar pattern was observed, with significant increases in intermolar width and arch circumference. Although the Modified Little’s Irregularity Index (LII) did not differ significantly between the groups, the intervention group showed a slight decrease in maxillary LII and a slight increase in mandibular LII, suggesting a possible trend toward improved anterior alignment stability in the maxilla.

This study indicated that performing occlusal adjustment during the first month after debonding-despite full-time use of an Essix retainer-was associated with significant increases in intercanine width, intermolar width, and arch circumference after six months. This observation can be explained by the unique biomechanical conditions of the early retention phase, during which the periodontal ligament remains in its initial remodeling stage and occlusal interdigitation has not yet fully stabilized. Eliminating premature contacts at this sensitive interval redirects settling from deflective pathways toward a more physiologic cusp-fossa trajectory, facilitating a redistribution of functional forces in the buccolingual dimension. Moreover, the semi-passive nature of the Essix retainer limits its ability to control transverse movements, allowing modified functional forces following adjustment to induce subtle buccolingual micro-drift. Although minimal at the level of individual teeth, these cumulative displacements can produce measurable arch-level changes of several millimeters.

Furthermore, the results of the present study align with the findings of Edman Tynelius et al. ^11^, who reported that much of the transverse dimensional change occurs within the first two post-treatment years, primarily as a result of natural settling. Occlusal adjustment appears to accelerate this physiological process, creating a form of “directed settling.” Adjustment-induced alterations in cusp morphology-changes in cusp height, inclination, or tip position-may also influence the anatomical landmarks used for measurements and contribute modestly to the observed increases. Taken together, these factors suggest that early occlusal adjustment can guide the pattern of occlusal adaptation, producing subtle yet meaningful transverse changes driven more by early retention biomechanics than by active orthodontic forces.

The rationale for occlusal adjustment as a post-treatment intervention lies in its ability to eliminate premature contacts, thereby reducing undesirable occlusal forces that may contribute to relapse ^7^. Several studies have emphasized the impact of occlusal discrepancies on tooth movement post-treatment. Baik et al. ^20^ highlighted that occlusal interferences could disrupt force distribution and compromise stability. Our observed increases in arch width and circumference may be indicative of a more balanced occlusal environment post-adjustment, potentially reducing the biomechanical stimuli for relapse.

Our results-specifically the lack of significant difference in LII-support the notion that occlusal factors may be necessary but not sufficient conditions for relapse prevention. Anterior alignment appears to be governed by a combination of dental, periodontal, muscular, and occlusal determinants.

Of particular relevance is the recent study by Marcos Fernando et al. ^21^, which evaluated occlusal adjustment in a non-extraction cohort over five years and reported no significant differences in LII or PAR scores. In contrast, our study specifically targeted high-risk extraction cases with a shorter follow-up, which may explain the observed differences in outcomes. Furthermore, both studies employed Essix retainers, which, as demonstrated by Farooqui et al. ^22^, may provide less effective long-term retention compared to fixed retainers. Consequently, the use of Essix retainers in our study might have moderated the detectable benefits of occlusal adjustment.

In conclusion, our findings suggest that selective occlusal adjustment may contribute to improved transverse arch stability following orthodontic treatment involving extractions. While the intervention appears to enhance arch width and circumference, its isolated effect on anterior alignment relapse remains inconclusive. Future research with larger sample sizes, extended follow-up durations, and comparative analysis of retention modalities is warranted to better delineate the role of occlusal adjustment in maintaining post-treatment stability.

## CONCLUSION

In this study, significant increases in intercanine and intermolar distances were observed in both the maxilla and mandible across intervention and control groups; however, these changes were significantly greater in the intervention group, indicating a more pronounced arch expansion following selective occlusal adjustment. Similarly, arch circumference changes were significantly larger in the intervention group compared to controls. Although Little’s irregularity index decreased in both groups, these changes were not statistically significant. A trend toward greater improvement in the maxillary irregularity index was noted in the intervention group, whereas in the mandible, a slight increase was observed without a significant difference. Overall, these findings suggest that selective occlusal adjustment may contribute to improved post-treatment stability by promoting favorable dimensional changes in dental arches, thereby potentially reducing orthodontic relapse.

### Limitations

The follow-up period was limited to six months post-debonding. Because relapse in arch dimensions and alignment may continue beyond this interval, long-term stability could not be fully evaluated. This study was conducted in a single orthodontic center, which may limit the external validity of the results, as differences in treatment protocols and retention strategies in other settings may influence the outcomes. Patient compliance with retention protocols (e.g., wear duration and appliance maintenance) was self-reported and may have been inaccurately recorded, which could have potentially affected the relapse measurements. Finally, the Modified Little’s Irregularity Index does not capture three-dimensional alignment changes; therefore, subtle rotations or vertical discrepancies might not have been detected.
